# Asking the right questions: Scoping studies in the commissioning of research on the organisation and delivery of health services

**DOI:** 10.1186/1478-4505-6-7

**Published:** 2008-07-09

**Authors:** Stuart Anderson, Pauline Allen, Stephen Peckham, Nick Goodwin

**Affiliations:** 1 Department of Public Health and Policy, London School of Hygiene and Tropical Medicine, London, WC1E 7HT, UK

## Abstract

Scoping studies have been used across a range of disciplines for a wide variety of purposes. However, their value is increasingly limited by a lack of definition and clarity of purpose. The UK's Service Delivery and Organisation Research Programme (SDO) has extensive experience of commissioning and using such studies; twenty four have now been completed.

This review article has four objectives; to describe the nature of the scoping studies that have been commissioned by the SDO Programme; to consider the impact of and uses made of such studies; to provide definitions for the different elements that may constitute a scoping study; and to describe the lessons learnt by the SDO Programme in commissioning scoping studies.

Scoping studies are imprecisely defined but usually consist of one or more discrete components; most commonly they are non-systematic reviews of the literature, but other important elements are literature mapping, conceptual mapping and policy mapping. Some scoping studies also involve consultations with stakeholders including the end users of research.

Scoping studies have been used for a wide variety of purposes, although a common feature is to identify questions and topics for future research. The reports of scoping studies often have an impact that extends beyond informing research commissioners about future research areas; some have been published in peer reviewed journals, and others have been published in research summaries aimed at a broader audience of health service managers and policymakers.

Key lessons from the SDO experience are the need to relate scoping studies to a particular health service context; the need for scoping teams to be multi-disciplinary and to be given enough time to integrate diverse findings; and the need for the research commissioners to be explicit not only about the aims of scoping studies but also about their intended uses. This necessitates regular contact between researchers and commissioners.

Scoping studies are an essential element in the portfolio of approaches to research, particularly as a mechanism for helping research commissioners and policy makers to ask the right questions. Their utility will be further enhanced by greater recognition of the individual components, definitions for which are provided.

## Background

Investigations under the title 'scoping studies' have appeared in the literature at regular intervals over a number of years. They have been used across a broad spectrum of academic disciplines and fields of study, including agricultural research [1,2], environmental studies and process engineering [3,4]. They have found particular utility in relation to public services, including education [5,6], housing and health care [7,8]. The literature in which they appear is international, and typically they consist of non-systematic reviews of the literature. However, the objectives set for scoping studies are diverse. The type and range of reports described as scoping studies are in reality extremely wide ranging, from reviews of qualitative studies to much more descriptive pieces.

Mays, Roberts and Popay suggest that 'scoping studies aim to map rapidly the key concepts underpinning a research area and the main sources and types of evidence available' [9]. In this review article we argue that the concept of the scoping study is itself now too broad to be of great utility without considerable further elaboration. We base our argument on a review of the literature in this area, and on our experience with commissioning research for the Service Delivery and Organisation Research Programme (SDO Programme) of the National Institute for Health Research in Great Britain. The SDO Programme has now commissioned a total of twenty four scoping studies.

The aim of this article is to review scoping studies commissioned by the SDO Programme in the light of the existing literature on this type of investigation. Its objectives are:

• to describe briefly the nature of the scoping studies that have been commissioned by the SDO Programme;

• to consider the impact of and uses made of such studies;

• to provide definitions for the different elements that may constitute a scoping study; and

• to describe the lessons learnt by the SDO Programme in commissioning scoping studies.

In addressing these objectives a number of the component elements of scoping studies are identified and defined. These include mapping the literature, policy mapping and conceptual mapping. Other common aspects of scoping studies, such as consultation exercises, are also described.

The problems of searching for qualitative research using electronic databases are well known to health service researchers, and the value of undertaking a preliminary scoping of the potentially relevant literature to assess the nature and distribution of relevant studies is fully recognised [9]. The studies that need to be identified tend to be extremely diverse in both their focus and design, and the problem of achieving both comprehensiveness and precision soon becomes intractable [10]. The review team needs to include a broad range of subject experts and to use a wide sweep of search terms [11].

There is now an extensive literature on the commissioning of research, extending from the setting of the research agenda to getting the findings of research used in practice both by health professionals and policy-makers [12,13]. However, much of the literature relates to priority setting in biomedical research rather than in health services research; and much of it emanates from the research institutes themselves [14,15]. Much has also been written about the need to commission research that is used, and about the assessment of how health research is used in policy-making [16-19]. There is now a substantial literature on establishing research priorities and the techniques available to assist with this, such as consensus development, a technique that has been used in several areas including critical care [20], on the interface between primary and secondary care [21], and in guideline development [22].

Rather less has been written about the processes by which the research questions to be addressed are arrived at in the first place – a subtly different process to priority setting since it implies an understanding of the more detailed investigative issues to be addressed as well as an understanding of the feasibility and nature of the research implied. Whilst funding bodies with a responsive mode rely heavily on researchers themselves to identify the questions to be addressed, those who commission research on specific themes or topics tend to rely on a broader range of sources. These include requests from other agencies, directors of national programmes, and syntheses of existing research literature. One further source that has received some attention is the views of service users [23]. Scoping studies are sometimes used to draw information from all these sources together to clarify the core research questions to be posed.

### The SDO Programme

The NIHR Service Delivery and Organisation Research and Development Programme (SDO Programme) was set up in 1999. It is now one of the constituents of the National Institute for Health Research, along with other programmes which include the Health Technology Assessment Programme and the Research for Patient Benefit Programme [24]. It is managed by a National Co-ordinating Centre (NCC SDO) based at the London School of Hygiene and Tropical Medicine.

The remit of the SDO Programme has recently been revised, but its core function has always been to commission research on service delivery and organisational aspects of health services. Its mission reads: 'The NIHR Service Delivery and Organisation Programme improves health outcomes for people by commissioning research and producing research evidence that improves practice in relation to the organisation and delivery of health care, and building capacity to carry out research amongst those who manage, organise and deliver services and improve their understanding of research literature and how to use research evidence' [25].

The SDO Programme's research agenda is determined by its Programme Board, and is informed by a variety of mechanisms that include listening exercises, discussions with other NHS organisations, and senior officials such as chief executives and national clinical directors. The role of listening exercises has been described elsewhere [23]. The SDO Programme is free to commission research into any aspect of health service delivery and organisation. However, additional funding may be made available to the SDO by the Department of Health for research in specific areas such as public health [26].

In developing new programmes of commissioned research the SDO Programme considers a wide range of factors, including how well particular concepts are understood and defined, what is already known in the area, the policy context, and the views of relevant stakeholders. Where information about this is lacking or limited the SDO Programme will consider commissioning a scoping study.

The SDO Programme commissioned its first scoping study in 2000. Since then some twenty four such studies have been commissioned, and final reports received. Some of these studies have subsequently been published in peer reviewed journals, and some of the researchers have described their experiences of carrying out such studies. The nature of the scoping studies commissioned has been revised and refined in the light of experience. This review describes the purpose, nature and impact of these studies, and draws lessons on the value and limitations of scoping studies for both commissioners and researchers.

### Existing definitions of scoping studies

As Arksey and O'Malley have found [27], definitions of scoping studies are few and far between. Mays, Roberts and Popay suggest that 'scoping studies aim to map *rapidly *the key concepts underpinning a research area and the main sources and types of evidence available' [28]. They 'can be undertaken as stand-alone projects in their own right, especially where an area is complex or has not been reviewed comprehensively before'. This definition suggests that scoping studies may be more than just non-systematic reviews of the literature; the exercise may or may not entail conceptual mapping, the mapping of policy documents and consultations with stakeholders, as well as mapping the literature.

Arksey and O'Malley demonstrate that this definition emphasises the need for breadth in the coverage of the available literature whilst leaving open the issue of depth of coverage according to the purpose of the review. They suggest that the type of scoping study that results will be very dependent on the aims set for it. They identified four common reasons why a scoping study might be undertaken:

• To examine the extent, range and nature of research activity in a particular area;

• To determine the value of undertaking a full systematic review;

• To summarise and disseminate research findings; and

• To identify research gaps in the existing literature [27].

These are broad aims, and clearly the term 'scoping study' can be little more than an umbrella term, having a variety of functions. This raises a further question: who decides whether a particular piece of work is a scoping study or not? The SDO Programme has generally described such work that it has commissioned as scoping studies. However, the researchers undertaking this work have not always described it as a scoping study in their final report and publications. Likewise others who have undertaken work not described as a scoping study in the commissioning brief have nevertheless described it as such in their final report or published papers.

### Nature of scoping studies commissioned by the SDO Programme

After six years of commissioning such studies we have undertaken a comprehensive review of the SDO Programme's experience in this area. The Programme's research database was examined to identify all projects that did not involve empirical research. Inclusion criteria were those which included scoping in the title, non-systematic reviews of the literature, reviews of concepts, consultation exercises specifically related to research agendas, and reviews of policy documents for indications of research priorities. Only projects where final reports had been accepted by 31 August 2007 have been included. By that date a total of 205 projects had been commissioned. Of these twenty four were classified as scoping studies. These are listed in Additional file 1.

The 24 scoping studies represent a great diversity of topic and they have varied greatly in breadth as well as depth. For example, scoping studies that have concentrated on analysing the broader landscape of a subject included an examination of the relationships between organisations [29], on organisational factors and performance [30], and on public health and outpatient services [31,32]. In-depth studies of a much narrower topic have included those on specialist rehabilitation for neurological conditions [33] and on measuring outcomes for carers of people with mental health problems [34]. In this review we discuss selected SDO scoping studies that illustrate some of the key points emerging from the review.

### Continuity of care scoping studies

The first scoping study to be commissioned by the SDO Programme was that on continuity of care. This had been identified as an important issue in health service delivery [35], but it was clear to the research commissioners that there was no clear understanding of what was meant by the term, or how it might be evaluated. The SDO Programme identified a need to commission a scoping study of continuity of care to provide definitional and conceptual clarity and so commissioned a limited non-systematic literature survey [36]. The research also identified theoretical approaches that could suggest new research directions, and sought the views of relevant patient groups. Their conclusions were presented in a final report, and the experience of working on this scoping study has been reported elsewhere by the researchers [37]. In this they emphasised the importance of having a team with a broad range of disciplinary backgrounds who could work quickly and flexibly, the importance of regular meetings with the research commissioners, and the need to agree the format of the final document with the commissioners at an early stage.

The continuity of care scoping study proved highly influential to the SDO Programme in justifying the value and purpose of commissioning a scoping study. In particular, it enabled definitional clarification of the contested and diffuse subject of continuity of care; identified key areas for future research; provided ideas for theoretical approaches that could be used in future research; and engaged with 'end users' to provide advice on what kind of research products might be useful in the 'real world'.

### Other early scoping studies

As other research areas were identified as candidates for scoping studies it became clear that different elements would be needed in each case. The second scoping study commissioned, a methodological scoping exercise on eliciting and assessing users' views on the processes of health care, necessitated both a literature review and a consultation exercise with stakeholders [38].

Access to health care services emerged as another important field for further research from the consultation exercise [35]. As with continuity of care, this was a concept with contested definitions and methods of evaluation, and the SDO Programme again identified this as a suitable candidate for a scoping study. It recognised that this would be an enormous undertaking, and opted to split the task into two parts; the first involved a review of the literature, and the second encompassed a conceptual mapping exercise, a policy review and a consultation exercise. The studies were undertaken by separate research teams, and were published as separate reports [39,40].

Subsequent scoping exercises have allowed for further clarification of both the purpose and process of this approach. Commissioning briefs for scoping studies have become more focused, the components of such studies often being commissioned separately from different research groups. Examples of these have been scoping studies of the healthcare workforce, those on service delivery aspects of e-health, and a number involving the identification of research priorities for patient- and carer-centred mental health services. A brief discussion of these follows.

### Workforce scoping studies

Workforce issues have been consistently near the top of the list of research priorities for the SDO Programme since its formation in 1999. There existed a considerable body of research on this topic published over several years, and a number of research commissioners, such as the Policy Research Programme of the Department of Health, had active programmes of work on human resource issues. For the SDO Programme the initial task was to identify the specifically service delivery and organisational aspects of this agenda, and to establish where the key gaps in knowledge and evidence were. The SDO remit on workforce issues was established following discussions with other commissioning bodies.

Enquiries quickly established that the evidence base in some areas (for example, skill mix in primary care) was quite strong, but in others was weak or unclear. It was clear that in total this was a large undertaking, and it was agreed that it could not reasonably be undertaking by a single team within a limited timescale. As a result the SDO Programme decided to commission three separate workforce scoping exercises, as follows:

• a review of the evidence around skill mix in secondary care;

• a review of the evidence on the relationship between workforce and health outcomes;

• a review of the evidence on the impact of local labour market factors on the organisation and delivery of health services.

As relative short and inexpensive pieces of work all three were offered to research collaboratives approved as rapid response teams. In the event it was clear that the specialist expertise required to undertake the review of local labour market evidence was not available within the teams, and this scoping exercise was advertised and awarded externally.

This exercise resulted in three extensive scoping reports on the state of knowledge relating to key aspects of the health workforce; in skill mix in secondary care [41], on the relationship between workforce and health outcomes [42], and on the impact of local labour market factors [43]. They demonstrated that research in this area was largely based on small case studies, was rarely underpinned by sound theory, and failed to constitute a cumulative body of knowledge. These scoping studies formed the basis of the SDO Programme's subsequent programme of research on the healthcare workforce. Some nine empirical studies have now been commissioned in this area.

### E-health scoping studies

In 2003 the SDO Programme Board identified e-health as an important area for future research. As with workforce, the scope of this field was considered too vast for any meaningful empirical research to be commissioned without first undertaking some kind of scoping study. As with workforce the first step was to identify the remit of the SDO Programme in this area; this was limited to service delivery and organisational aspects, including workforce and change management issues, and excluded any evaluation of e-health technologies themselves.

The second step was to identify what elements of a scoping exercise needed to be included. The decision taken was that there was in fact a need for a comprehensive mapping and scoping exercise. This would involve conceptual mapping of the field, a review of the literature, a review of the e-health policy context and a consultation exercise with stakeholders.

The third step was to consider whether this should be commissioned as a single scoping exercise, as four separate studies, or some combination of this. In the event two scoping exercises were commissioned: the first examined the e-health context and undertook a consultation exercise with stakeholders [44]; the second provided the conceptual map of the field and included a review of the literature [45]. The reports of these two scoping exercises provided a foundation for future empirical research in this area commissioned by the SDO Programme.

### Research priorities scoping studies

Priorities for research in specific areas are identified in a variety of ways by many different groups, including researchers, advocacy groups, policy makers and practitioners. In order to take research agendas forward an essential first step is to undertake a synthesis of these various research priorities, to rank them, and to identify the most appropriate commissioners for the research.

Regardless of their stated objectives the reports of scoping studies very often include a statement of research questions and topic areas in need of investigation. In fact an initial review of SDO scoping studies was carried out at the end of 2002 [46]. At that stage six reports had been received. The purpose of that review was to identify research questions and topics that had been identified in the scoping studies. Although the scoping studies varied considerably in purpose, nature and content all generated suggestions for further research.

The SDO Programme has now commissioned a number of scoping studies specifically designed to identify research priorities. The first was designed to identify research priorities in nursing and midwifery service delivery and organization [47]. The report of this study formed the basis of the nursing and midwifery research programme over the next few years. A more recent study resulted from the need to identify research priorities for patient- and carer-centred mental health services. The decision taken this time was that, although there was no need for a conceptual mapping of the field, there was a need for a targeted review of the literature and a review of mental health policy documents to identify all the research questions and priorities raised. There was also a need for a consultation exercise with stakeholders.

Again, two separate scoping studies were commissioned, with a separate one for the consultation exercise. Reasons for separating these activities include recognition that the research skills needed are sometimes to be found in different research groups. In the event both scoping studies were awarded to the same institution, which enabled full cooperation between the groups. It also allowed for the easy combining of the findings of each study into a single overview report. Thus three final reports were received; a synthesis of the literature and policy documents [48]; a report on the consultation exercise [49]; and an overview report [50]. More recently a scoping and consultation study has been commissioned to establish the research priorities in learning disabilities [51]. On this occasion the scoping study was commissioned as a single study.

### The limitations of scoping studies

As the following sections to this article will show, commissioning scoping studies can result in a range of benefits on a number of fronts. However, our experience with scoping studies points to a number of potential limitations. One of the most obvious of these is that scoping studies are often commissioned in a 'rapid response' mode to enable commissioners to develop subsequent commissioning briefs quickly. However, scoping studies often require 'sense-making' across fields of enquiry that are complex and which lend themselves to interpretation through many academic and theoretical disciplines. In these cases the ability to commission a scoping study of an appropriate quality that covers the breadth and depth of the topic may be no more speedy than a more traditional systematic literature review.

As the examples of the e-health and workforce scoping studies showed, the specialist knowledge required to adequately map a subject was not always found in a single individual or research team but required multi-disciplinary input through separate studies, some procured through competitive tender. This meant that a key role had to played by the commissioner in co-ordinating the scoping studies (to meet the commissioner's common objectives and time-scales) and in managing the trade-off between the need for a rapid appraisal of a subject and the necessary quality required in the scoping studies themselves. An associated limitation is also present in the potential bias in the perception and interpretation of a subject due to a researcher's prevailing academic discipline and research interests. To avoid such bias, scoping studies themselves often needed quite detailed commissioning briefs and a peer-review process of the final product.

Another limitation worth noting is the status of the scoping paper within the academic community. The value of working on developing a scoping paper, for example in terms of time and financial remuneration, was potentially problematic, especially since the nature of the scoping study as a non-systematic review might not enable peer-reviewed academic articles to be developed. The fact that many scoping studies became translated into peer-reviewed products, or had impact in other ways (see below), implies that scoping studies have, and should, act as key sources of research findings for dissemination but that there are also associated responsibilities for researchers and commissioners to ensure that the nature and limitations to scoping studies are reported.

### Impact of SDO Programme scoping studies

A recent review of the impact of research commissioned by the SDO Programme has demonstrated the many different types of impact that result [52]. An important feature of many scoping studies commissioned by the SDO Programme is the extent to which they identify topic areas for future research; however, this is rarely the only consequence of a scoping report. Scoping studies frequently have an impact which goes far beyond that which might be expected based on their cost and length; this includes impact on both policy and practice [53]. The SDO Programme frequently judges scoping studies of sufficient merit to justify publication of a briefing paper, and they are often published in peer reviewed journals in their own right.

### Briefing papers or research summaries

Reports of four scoping studies commissioned by the SDO Programme have so far resulted in briefing papers or research summaries published by the SDO. These are briefing papers on services to support carers of people with mental health problems and on achieving high performance in health care systems [54,55]. Subsequently research summaries have been published on the role of nurses, midwives and health visitors in improving children's health and on the role that primary care can play in reducing demand on hospital outpatient departments [56,57]. Briefing papers and research summaries provide a mechanism for presenting the results of scoping studies to a wide health service audience in an easy to read and accessible way.

### Peer reviewed publications

The final reports of all the scoping studies appear on the SDO Programme's website. However, in addition researchers are encouraged to secure publication of edited versions of the reports in peer reviewed journals, and to write about their experiences of conducting them. To date edited versions of two of the scoping study final reports have appeared in peer reviewed journals. These are that on services for carers of people with mental health problems [58], and the scoping study on identifying research priorities in nursing and midwifery service delivery and organization [59]. This ensures that the results of scoping studies are available to the wider academic community.

### Books

Scoping studies are often substantial pieces of work in their own right. Outside the health field publication in book form is a common mechanism for publicizing the results of a scoping study. One of the scoping studies commissioned by the SDO Programme, that on access to health care services, has subsequently resulted in an important book and is now a standard work in this area [60].

### Further research

For the SDO Programme an important aim in commissioning scoping studies has been to identify important questions for future research. Indeed, this was the primary propose of commissioning many of the scoping studies. They have been used to identify research priorities in nursing and midwifery service delivery and organization, and for patient- and carer-centred mental health services. The former established the basis of the SDO's nursing and midwifery research programme over the following years. The scoping studies on workforce issues and on e-health have likewise been instrumental in defining the research agendas in these areas. The continuity of care scoping study led directly to the commissioning of five large empirical studies in this area, and the conclusions of these have themselves been synthesised into an overview report [61].

### Defining the elements of scoping studies

The scoping studies commissioned by the SDO Programme constitute a diverse group of studies, often containing a number of different elements. Nevertheless they generally consist of one or more of four major components; a literature map, a conceptual map, a policy map and a consultation with stakeholders. These components constitute a large proportion of all publicly accessible scoping studies in health care and beyond. We believe that it is now necessary to develop more robust definitions for these components, and we will consider each in turn.

We have considered the appropriate terminology for these components at some length. The term 'mapping' has a specific meaning in geography and in bibliometric analysis (especially library based studies designed to illustrate the historical development of literatures). In the context of the discussion here it could have one of several competing meanings. Policy mapping in particular is a widely used term, and is related to Elmore's 'backward mapping' in policy analysis. However the term 'mapping' is already referred to in the literature in many other ways to describe a range of activities. Nevertheless, we take the view that, in relation to scoping studies, 'mapping' provides a useful and meaningful shorthand for what is described in the components described.

### Literature mapping

The most common form of scoping study is a map of the relevant literature. These vary in scope from general accounts of the literature to studies that are just short of systematic reviews. Literature scoping studies often also involve the syntheses of findings from different types of study. There is now a vast literature on reviewing the literature [62], including systematic reviews [63], which provide explicit definitions of what a literature review is and how to conduct it [64]. There is also a growing literature on techniques for synthesising complex evidence [65], including that from narrative sources [66].

We believe that there is a clear case for distinguishing between non-systematic reviews of the literature and literature mapping exercises. Mapping the literature usually provides the greatest challenge to those tasked with undertaking these scoping studies. In almost all areas the literature is vast, diffuse and of variable quality. Literature mapping aims to provide an initial indication of the location of the literature relating to a particular issue and to identify its overall size. Its objective is to map out the literature as it stands, without any immediate plan to review it systematically. This means plotting it out in time (last five years or longer?), space (UK, USA or whole world?) source (mainly peer reviewed journals or grey literature?) and origin (social science academics or health professionals?). It is therefore a preliminary stage prior to a full literature review.

A good literature map spells out the origins of work on this topic and gives a good feel for its chronological development. It gives a good account of where this work has been carried out and why. For example, is most of the relevant literature American, Australian or French? It also gives a good account of who has done this work; is it doctors, psychologists, economists or sociologists? What was the order in which each group became involved? Other key questions that literature mapping can address are: Can large parts of it largely be dismissed because of fundamental flaws in the methodology? What are the key areas where good evidence appears to be available?

Answering these questions simply provides the background to mapping the literature itself. As well identifying where the literature is the map needs to give some indication of strengths and weaknesses in the literature. A literature map can therefore be defined as *'a scoping study designed to provide an initial indication of the size and location of the literature relating to a particular topic as a prelude to a comprehensive review of the literature.'*

### Conceptual mapping

Concept mapping is recognised as a separate exercise in its own right, and has been used in policy evaluation for some years [67], although no clear definition of a concept map has been described. A conceptual map generally explores the terminology in use with regard to a particular topic. It lists what the key terms are (for example, concordance, adherence and compliance) along with any supplementary terms (for example, non-intentional adherence) It is designed to elicit how particular terms are used, by whom and for what purpose. There may, for example, be differences in the meanings attached to words or phrases by different disciplines. Not infrequently the same word is used to represent very different concepts by different disciplines. This clearly makes literature searching a hazardous activity.

Examples of scoping studies commissioned by the SDO Programme that have included conceptual mapping include the continuity of care and access studies [68,69]. Conceptual mapping has often been an important element within broader literature mapping exercises. For example, the concepts of adherence, compliance and concordance in relation to medicine taking have been treated in a number of ways, and a scoping study was commissioned to make sense of the various uses of these terms [70]. Likewise, the phrase 'fallers clinics' has been used in many different ways, and a scoping study was commissioned to clarify the situation with a view to additional research being undertaken in this area [71].

A concept map can therefore be defined as *'a scoping study designed to establish how a particular term is used in what literature, by whom and for what purpose.'*

### Policy mapping

Policy in health care has developed over many years, and sources for it are often to be found in a large number of separate policy documents. Not all of these are published by government; important policy statements are to be found in documents of health agencies such as the Health Care Commission and the National Institute for Health and Clinical Excellence (NICE), the Royal Colleges, and in European legislation. Policy mapping exercises are frequently needed to trace all relevant documents. Policy initiatives often have implications for research, and funding agencies need to map such documents for this purpose [72].

A policy map identifies the main documents and statements from government agencies and professional bodies that have a bearing on the nature of practice in that area. It is usually necessary to make a clear distinction between economic policies that impact the service and those of a specifically health policy nature. These include policies which are disease specific (for example, diabetes and Alzheimer's disease), those that are client group specific (children, older people) and those that are system wide (mental health). Other relevant ones relate to specific health service staff groups, such as nurses, general medical practitioners and pharmacists.

The documents issued by the Department of Health normally covered in policy mapping include National Service Frameworks, other health related policies include directives from the Health Commission, the Audit Commission and Monitor in relation to NHS foundation trusts. Documents issued by other government departments may also be relevant. So too might those issued by Royal Colleges and other professional regulatory authorities. Examples of scoping studies commissioned by the SDO Programme which incorporate a policy mapping exercise include the development of research agendas for mental health, learning disabilities, and access to heath care.

A policy map can therefore be defined as *'a scoping study designed to identify the main documents and statements from government agencies and professional bodies that have a bearing on the nature of practice in that area.'*

### Stakeholder consultations

The importance of consulting with all stakeholders with an interest in the development of a service or establishment of a research agenda is now well recognised, and techniques for doing so well established. One of the early tasks of the SDO Programme was to carry out a national listening exercise in 2000 [73], and this reported in the same year [74]. This exercise was repeated in 2002 [75], when it was found that the priorities of stakeholders had changed slightly, reflecting changes in the major concerns in the health service at the time [76].

The role of consultation with stakeholders in the setting of priorities for applied health services research has been discussed at length from a joint English and Canadian perspective [77]. The authors concluded that 'listening exercises' were a useful way of helping to set the agenda for user-driven research, and were a useful addition to the priority-setting toolbox. Moreover, the experience of the SDO Programme also suggests that 'informed' stakeholders (particularly policy makers or other 'users') may also play a key role as consultant 'subjects' of a scoping study, particularly in helping to define relevant questions and/or to peer-review and validate the end project [19]. However, there appears to be no substitute for investigator-initiated processes for setting priorities, as stakeholders can rarely identify anything more than broad themes for which more research is needed. They nevertheless play a useful part in promoting stakeholder involvement in research and in helping commissioners to subsequently target research questions that may lead to more 'usable' research products [16].

Stakeholder consultations therefore do not constitute scoping studies in their own right, but they do have an important part to play in scoping studies concerned with the identification of research priorities, in helping to target research questions, and in validating the outcomes of scoping studies through peer-review.

### Other elements of mapping and scoping

Although policy, literature and concept mapping, along with consultation exercises, account for the vast bulk of scoping studies, they by no means account for them all. This list is not comprehensive, and published scoping studies have embraced a number of other issues. Scoping studies are often commissioned to inform researchers, policy makers and research commissioners about what the key gaps in knowledge are. This may include methodological research, such as the mapping of outcome measures. The SDO Programme has commissioned such methodological scoping studies, including one on eliciting and assessing users' views on the processes of healthcare [78].

This list is in no way comprehensive or restrictive. Indeed, the SDO Programme has itself commissioned scoping studies that do not easily fit within the categories described above, such as its study to conceptualise the contribution of nursing, midwifery and health visiting to child health services [79]. For scoping studies that include several types of mapping another important element is the synthesis of the different strands of the exercise.

### Lessons learned by the SDO Programme in commissioning scoping studies

The experience of commissioning scoping studies has been reviewed informally by the SDO Programme at regular intervals. An early review found that the policy of commissioning scoping reports as an initial stage in commissioning research had proved useful [80].

Two of the teams who have carried out scoping studies commissioned by the SDO Programme (those on continuity of care and on services to support carers of people with mental health problems) have described their experience of undertaking scoping studies in the academic literature [81,82]. The latter paper explored some of the methodological issues raised by scoping studies.

The SDO Programme now has substantial experience of both the strengths and weaknesses of scoping studies, the circumstances under which they are appropriate, and the balance that needs to be made between prescription and flexibility. They are an extremely valuable tool, and in many cases are an essential prerequisite to more detailed empirical research. They provide the opportunity to map a wide range of literature, and allow researchers to identify where gaps in our knowledge may lie, along with any particularly inventive or innovative approaches that may have been missed. A number of key lessons have been learned by the SDO Programme from this experience, and these are now considered further [80].

### The importance of context

A review of scoping reports has demonstrated that simply presenting a map of the current research evidence is not of itself enough to ensure that any research subsequently commissioned by the SDO Programme would be sufficiently relevant to current activities and concerns in the National Health Service (NHS). Early scoping reports had important information missing concerning the relationship between the formal evidence and the current health care context of the NHS.

Subsequent scoping studies have included an analysis of the current key issues and concerns relating to the issue under investigation. This often involves an examination of local implementation of national policy developments, and any specific concerns at the local level in the NHS. Under these circumstances a scoping study may need to assess any unpublished regional or local research that may have been carried out.

### Multi-disciplinary scoping teams

A common feature of scoping studies commissioned by the SDO Programme has been the need to engage researchers from a wide range of academic disciplines. Literature relevant to the topic under investigation is often dispersed, and it may be necessary to include researchers with epidemiological and systematic review experience as well as sociologists, psychologists and anthropologists.

This can create its own problems, as researchers from very different theoretical perspectives often have difficulty in working together, and this is sometimes reflected in the final report submitted. Time and effort is required to integrate the separate findings and recommendations for further research into a coherent final report.

### Timings and meetings

The time allowed for the conduct of a scoping study is a matter of balance. Our experience is that scoping studies that are too rapid tend to be unsatisfactory. The original continuity of care scoping exercise was completed in three months; experience suggests that such a tight time scale prevented a more considered and comprehensive response, and that typically six months is a more appropriate time.

Experience also suggests that regular meetings between the researchers and the research commissioners are essential to ensure that the team delivers what is required. An initial meeting needs to be held at the commencement of the work, and another should be held at the half way stage. It has also proved invaluable to hold meetings at the report writing stage, with the research commissioners commenting on early drafts, to ensure that the final report fulfils expectations.

### Recommendations for further research

The primary purpose of many of the scoping studies commissioned is to inform research commissioners about what needs to be done next. This aim needs to be made very explicit. Several early scoping reports did not give sufficiently clear recommendations for further research; there was a lack of connection between the recommendations made and the content of the literature review contained in the report.

Scoping studies are indicative and suggestive rather than definitive and prescriptive. It is important to allow researchers some discretion and freedom to conduct the scoping study as they think fit, although this is not always an easy balance to get right. Informal approaches to other researchers in the field may be helpful. The reports of scoping studies tend to be of considerable interest to other researchers in the field, who may wish to develop their own research programmes around the findings.

## Conclusion

This paper has described, from the perspective of the commissioning body, the nature of the scoping studies that have been commissioned to date, and how they have contributed to taking the research commissioning process forward. From the evidence and experience of the twenty-four scoping studies considered in this review, an emergent set of key criteria for commissioning a scoping paper has developed [see Additional file 2].

Scoping studies have been found to be particularly useful in identifying the services available for dispersed and vulnerable groups. An early scoping study of this type was that on services to support the carers of people with mental health problems [83], and more recently a scoping study has been undertaken of generalist services available to people at the end of life [84].

Scoping studies can potentially have a number of specific and discrete components, such as literature and policy mapping. In considering the need for a new scoping study, the value and relevance of each of these components needs to be considered and eliminated where appropriate, according to the declared aims of the scoping exercise. In developing new research areas consideration is given to whether or not a preliminary conceptual mapping exercise is needed. Recent examples where this was judged not to be the case were self care and public health, where alternative approaches to the development of research agendas were taken.

The reports of scoping studies are often important research outputs in their own right. Evidence suggests that they can indeed have relevance far beyond informing the SDO Programme itself and other funders about future research priorities. Scoping studies have a variety of audiences, and care needs to be taken in the interpretation of their findings.

Clearly, scoping studies have a wide range of uses and take a great variety of forms. The phrase conveys a general idea but little more. Its use across a broad range of disciplines will doubtless continue, and its utility seems assured; there is no obvious alternative to the description of this diverse range of investigations as scoping studies.

What would seem both helpful and necessary, we suggest, is that where a more precise and focused definition is possible, such as literature mapping, policy mapping and conceptual mapping, this should be used; and that scoping studies which are non-systematic reviews of the literature should be described as such. Scoping studies conceptualise areas of research and other issues within their historical and cultural constraints. In relation to policy the aim is to relate research knowledge and issues within a contemporary policy context. Similarly, consultations with stakeholders identify current practice issues. Scoping studies are, therefore, concerned with contextualising knowledge in terms of identifying the current state of understanding; identifying the sorts of things we know and do not know, and then setting this within policy and practice contexts.

What we can say is that, whatever the stated aims and objectives of a scoping study, the ultimate aim of all scoping studies is to help research commissioners, health service managers, policy makers and researchers to ask the right questions.

## List of abbreviations used

NCCSDO: National co-ordinating centre for the NIHR service delivery and organisation programme; NHS: National health service; NIHR: National institute for health research; NICE: National institute for health and clinical excellence; SDO: Service delivery and organisation programme.

## Competing interests

SA, PA, NG and SP are academic staff of the NIHR Service Delivery and Organisation Programme.

## Authors' contributions

SA developed and wrote the article and prepared Additional file 1. All authors commented on later drafts. SP led the work on the impact of the SDO Programme's scoping studies. NG led on reviewing the role of scoping studies in influencing policy makers and practitioners, and prepared Additional file 2. PA led on the review of commissioning research that is used.

## Supplementary Material

Additional file 1 Service delivery and organisation research programme scoping studies 2000 to 2006. Lists the principal investigators, titles and component parts of the first twenty four scoping studies commissioned by the SDO Programme. Click here for file

Additional file 2 Key criteria for commissioning a scoping study. Lists eight key research objectives for which the commissioning of a scoping study might be appropriate. Click here for file
