# Supplementary material for: Asking the right questions: Scoping studies in the commissioning of research on the organisation and delivery of health services
Source: Health Res Policy Syst. 2008 Jul 9;6:7. doi: 10.1186/1478-4505-6-7 (PMC2500008; doi:10.1186/1478-4505-6-7)
Supplement: Additional file 1 — Service delivery and organisation research programme scoping studies 2000 to 2006. Lists the principal investigators, titles and component parts of the first twenty four scoping studies commissioned by the SDO Programme. [file 1478-4505-6-7-S1.doc]

**Project Principal investigator Year Title Con- Liter- Pol- Consult-**

**ceptual ature icy ation**

**mapping mapping mapping exercise**

SDO/02 Professor George Freeman 2000 Continuity of care scoping exercise √ √ x x

SDO/03 Professor Paula Nicolson 2000 Eliciting and assessing users' views on the processes of x √ x √

health care: a methodological scoping exercise

SDO/05 Dr Martin Gulliford 2000 Access to health care services: a scoping exercise x √ x x

SDO/12 Dr Rebecca Rosen 2000 Access to health care: taking forward the findings √ x √ √

SDO/15 Dr Hilary Arksey 2001 Mental health scoping exercise: research on services to x √ x √

support carers for people with mental health problems

SDO/19a Professor Ewan Ferlie 2002 Relationships between health care organisations: a critical x √ x x

overview of the literature and a research agenda

SDO/20 Professor Fiona Ross 2002 Identifying research priorities in nursing, midwifery service x √ x √

delivery and organisation: a scoping exercise

SDO/50 Professor Trevor Sheldon 2003 Workforce and health outcomes: a scoping exercise x √ x x

SDO/51 Professor Roy Carr-Hill 2003 The potential for improving the effectiveness of the workforce x √ x x

in secondary care: what is the evidence? A scoping exercise

SDO/52 Professor Robert Elliott 2003 The impact of local labour market factors on the organisation x √ x x

and delivery of health services: a scoping exercise

SDO/55 Professor Rod Sheaff 2003 Organisational factors and performance: a scoping exercise √ √ x x

SDO/59 Professor Ray Jones 2003 E-health policy context and consultation with stakeholders x x √ √

SDO/60 Dr Claudia Pagliari 2003 Literature review and conceptual map of the area of e-health √ √ x x

SDO/68 Professor Alison While 2004 A scoping exercise: the nursing, midwifery and health visiting x √ x x

contribution to child health services

SDO/76 Professor Rob Horne 2004 Concordance, adherence and compliance in medicine taking: √ √ √ x

a scoping exercise

SDO/81 Professor Tom Burns 2004 Measuring outcomes for carers for people with mental health √ √ x x

problems

SDO/82 Professor Martin Roland 2005 Outpatient services and primary care x √ x x

SDO/85 Ms Angela Greatley 2005 A synthesis of literature and policy documents for patient and x √ √ x

carer-centred mental health research priorities

**Project Principal investigator Year Title Con- Liter- Pol- Consult -**

**ceptual ature icy ation**

**mapping mapping mapping exercise**

SDO/86 Ms Angela Greatley 2005 Patient and carer-centred mental health services research x x x √

priorities: a consultation exercise with relevant stakeholders

SDO/132 Dr John Gladman 2006 Specialist rehabilitation for neurological conditions: a literature x √ x x

review and mapping study

SDO/139 Professor Sallie Lamb 2006 Scoping exercise on fallers' clinics x √ x √

SDO/143 Professor Irene Higginson 2006 A scoping exercise: generalist services for people at the end x √ x √

of life

SDO/150 Professor David Hunter 2006 Scoping study of the public health system In England x √ x x

SDO/152 Dr Ruth Townsley 2006 Shaping our future: a scoping and consultation exercise to establish x √ x √

research priorities in learning disabilities for the next ten years
