# Supplementary material for: Asking the right questions: Scoping studies in the commissioning of research on the organisation and delivery of health services
Source: Health Res Policy Syst. 2008 Jul 9;6:7. doi: 10.1186/1478-4505-6-7 (PMC2500008; doi:10.1186/1478-4505-6-7)
Supplement: Additional file 2 — Key criteria for commissioning a scoping study. Lists eight key research objectives for which the commissioning of a scoping study might be appropriate. [file 1478-4505-6-7-S2.doc]

**Additional file 2: key criteria for commissioning a scoping study**

| The decision to commission a scoping study should be based on an assessment of its value in meeting the following objectives:   - To clarify conceptual understanding of a topic where definitions are unclear or disputed; - To map and make sense of the extent, range and nature of research undertaken in a particular area. This might include the mapping of concepts, policies, evidence, and/or user views either separately or in combination; - To identify the strengths and weaknesses of the research base; - To identify gaps in research knowledge that need filling; - To determine the value of undertaking further systematic reviews or empirical research; - To advise on and make justification for further research studies and the core questions that need to be answered; - To develop methodological ideas and/or theoretical approaches best suited for future research studies of a particular topic; - To act as a source of research findings that can be disseminated to a wider audience. |
| --- |
